# Identification of differentially expressed circRNAs in prostate cancer of different clinical stages by RNA sequencing

**DOI:** 10.1038/s41598-023-48521-7

**Published:** 2023-12-01

**Authors:** Xing Wang, Ruizhen Huang, Juhui Yu, Fei Zhu, Xiaoqing Xi, Yawei Huang, Chiyu Zhang, Honglin Hu

**Affiliations:** 1 Department of Urology, The Second Affiliated Hospital of Nanchang University, No. 1 Minde Road, Nanchang, 330006 Jiangxi Province People’s Republic of China; 2 Northeast Yunnan Regional Center Hospital, Zhaotong, 657000 China; 3 Department of Urology, People’s Hospital of Wuyuan County, Shangrao, 33320 China

**Keywords:** Cancer, Genetics, Oncology

## Abstract

Circular RNAs (circRNAs) are linked to cancer, but it's still not clear what role they play in prostatic cancer. Through high-throughput sequencing, the goal of this study was to compare how circRNAs are expressed at different stages of prostate cancer. 12 patients attending the Department of Urology at the Second Affiliated Hospital of Nanchang University between June 2020 and October 2021 were used for RNA sequencing, and 14 patients were used for real-time fluorescent quantitative PCR (qRT-PCR). The expression profiles of prostate cancer circRNAs were constructed by sequencing with the help of next-generation high-throughput sequencing technology, and the differentially expressed circRNAs were analyzed by targeting microRNA (miRNA) loci and Gene Ontology (GO) enrichment analysis and Kyoto Encyclopedia of Genes and Genomes (KEGG) enrichment analysis of the genes from which circRNAs originated. Finally, the expression of target circRNAs in two prostate tissues was verified by qRT-PCR. Following high-throughput sequencing, 13,047 circRNAs were identified, and 605 circRNAs with significant differential expression were identified, of which 361 circRNAs were up-regulated, and 244 circRNAs were down-regulated. Analysis of circRNA-originated genes using GO and the KEGG enrichment analysis showed that circRNA host genes can regulate and influence multiple signaling pathways in prostate cancer with important biological functions. And the circRNA–miRNA network was constructed. The highest number of differentially expressed circRNA-binding miRNAs were: hsa_circ_000 7582 (52), hsa_circ_000 6198 (37), hsa_circ_000 6759 (28), hsa_circ_000 5675 (25), and hsa_circ_000 2172 (22). Moreover, we further screened out the circRNA (hsa_circ_0005692) that was significantly differentially expressed and common to all groups and verified by qRT-PCR that the expression of the target circRNA (hsa_circ_0005692) was significantly downregulated in prostate cancer compared with benign prostatic hyperplasia (BPH) tissues.

## Introduction

Prostate cancer (PCa) is now the second most common male malignancy worldwide and the second leading cause of cancer-related deaths in men^1^. Because prostate cancer is difficult to detect early, most patients are already at an advanced stage when seen, missing the best time for prostate cancer treatment^2^. Even with treatment, some patients still inevitably progress to castration-resistant prostate cancer (CRPC), with limited and uncertain treatment outcomes. Therefore, early diagnosis has an essential role in the prognosis of prostate cancer. Prostate-Specific Antigen (PSA) is a standard specific serum tumor marker for screening PCa. However, it has a high false-positive rate^3^. Therefore, there is an urgent need to explore tumor markers capable of early detection and diagnosis of prostate cancer. With the rise of next-generation sequencing technology, circRNAs were implicated not only involved in cellular physiological functions, but also in various human pathologies including cancer. It was found that circRNAs are aberrantly modulated in human cancer tissues^4^. Numerous studies have shown that circRNA is involved in the cell cycle, proliferation, migration, invasion, and metastasis of many tumors, such as oral squamous cell carcinoma, bladder cancer, gastric cancer, colorectal cancer, and prostate cancer^5^. It can be detected in common body fluids such as human blood and urine and is not easily degraded by nucleic acid exonucleases. These properties make cyclic RNA have the potential to become a biomarker^6^. Although liquid biopsy is a convenient and promising method, it must be complemented with invasive tissue biopsy to better guide disease diagnosis and treatment. Therefore, it is essential to study circRNAs in Pca tissues. This study aimed to investigate the expression profiles of circRNAs in prostate tissues of patients with different clinical stages of prostate cancer and BPH patients, perform preliminary functional prediction and analysis of differentially significantly expressed circRNAs, and identify new biomarkers for the assessment of prostate cancer. It is expected to add a new dimension to the study of molecular mechanisms of prostate cancer and will also provide a theoretical basis for the diagnosis and treatment of prostate cancer.

## Materials and methods

### Collection of samples

Human PCa and BPH specimens were collected from 12 PCa patients who underwent radical prostatectomy and electrical transurethral prostatectomy at the Department of Urology (The Second Affiliated Hospital of Nanchang University, Nanchang, Jiangxi, China) from June 2020 to October 2021 (Table 1). This study was approved by the Ethics Committee of the Second Affiliated Hospital of Nanchang University. Each patient gave written informed consent prior to participation in this study. Fresh tissues were frozen in liquid nitrogen immediately before use and stored at − 80 °C. Inclusion criteria: (1) patients with prostate cancer had a puncture biopsy and postoperative pathological diagnosis of prostate cancer, and patients with prostatic hyperplasia had a postoperative pathological diagnosis of benign prostatic hyperplasia. (2) No radical surgery, radiotherapy, endocrine therapy, and related biological agents were received. (3) Exclude other systemic primary oncological diseases and the presence of severe organ insufficiencies such as heart, liver, and kidney. Patients included in the study were staged according to the clinical stage according to the TNM staging criteria established by the International Union Against Cancer^7^, and TNM staging was performed according to clinical data such as imaging and pathology. A total of 12 patients with prostate cancer and BPH were then selected and divided into four groups of three cases each, which were labeled as (1) Prostate hyperplasia group, (2) Early limited group, (3) Local progression group, (4) Advanced metastasis group. The prostatic hyperplasia group included 3 patients with benign prostatic hyperplasia; the early metastasis group included 3 patients with TNM stage T_1_ and T_2_ prostate cancer; the local progression group included 3 patients with TNM stage T_3_; the advanced metastasis group included 3 patients with TNM stage T_4_ prostate cancer. The TNM was classified as T_4_. The above 12 patients were used to undergo high-throughput sequencing for circRNAs related studies, and their TNM staging information is shown in Table 1 below.

**Table 1:** Information on 12 patients who underwent high-throughput sequencing.

Patients with number	Age (years)	Prostate TMN staging
S1-1	66	T2N0M0
S1-2	66	T2N0M0
S1-3	64	T2N0M0
S2-1	70	T3bN1M0
S2-2	70	T3aN0M0
S2-3	73	T3bM0N0
S3-1	64	T4N0M1
S3-2	63	T4N0M0
S3-3	63	T4N0M1
S4-1	67	BPH
S4-2	79	BPH
S4-3	74	BPH

Another 14 prostate tissue specimens from patients with prostate cancer and BPH were collected for quantitative real-time fluorescence PCR (qRT-PCR). They will be divided into two groups: experimental group: 10 cases of prostate tissue from patients with prostate cancer; control group: 4 cases of prostate tissue from patients with benign prostatic hyperplasia.

### RNA extraction and quality assurance

Total RNA was extracted from the test samples by Trizol method, and the samples were firstly analyzed for DNA contamination and integrity by agarose gel electrophoresis; then the RNA concentration and purity (OD260/280) were detected by the NanoPhotometer® spectrophotometer (IMPLEN, CA, USA), and if the OD260/280 values were in the range of 1.8–2.1, the RNA purity was qualified; next, Agilent 2100 was used to detect the purity and integrity of the total RNA (Agilent Technologies, CA, USA). The sequencing of the test RNA samples was entrusted to Shanghai Jikai Gene Technology Co.

### Library construction, quality checking and sequencing

5 µg of each sample was prepared for library preparation. Ribosomal rRNA was removed using the Ribo-zero™ rRNA Removal Kit (Epicentre, USA), then eliminated linear RNA using RNase Rase (Epicentre, USA). The remaining circRNA was broken into short fragments of 250–300 bp, which were used as a template to synthesize the second strand of cDNA using random oligonucleotides as primers to synthesize the first strand of cDNA, followed by dNTPs (dUTP, dATP, dGTP, dCTP) as raw materials to synthesize the second strand of cDNA. The purified double-stranded cDNAs were end-repaired, A-tailed, and sequenced, and AMPure XP beads were used to screen 350–400 bp cDNAs in length (Beckman Coulter, Beverly, USA). cDNAs containing U were degraded with USER Enzymes (NEB, USA), and PCR amplified to obtain libraries. The fragment size and purity of the library were checked using Agilent 2100 to ensure the quality of the library. Illumina PE150 double-end sequencing was performed after passing quality control.

### Bioinformatic analysis

1. Assessing the quality of sequencing data: including the statistics of data volume, comparison rate, and sequencing error rate, the primary process is to remove the low-quality data and get the high quality and standard data, called "clean reads."
2. Information mining and analysis: The obtained CircRNA "clean reads" were compared to the reference genome, and then the circRNAs were identified by CIRI^8^ and find_circ^9^, and the results of both software were combined to perform qualitative and quantitative analysis of circRNAs. The differentially expressed circRNAs were identified using DESeq2^10^ based on the negative binomial distribution. In this experiment, a differential ploidy of twofold or more and a p-value of less than 0.05 were set as the screening conditions for differential RNAs. The differentially expressed circRNAs were subjected to enrichment analysis such as GO enrichment of source genes, KEGG enrichment analysis^11^ and prediction of miRNA binding sites and miRNA binding site prediction, etc.
3. CircRNA targeting miRNA loci analysis: CircRNA mainly exerts their biological function by binding to miRNA and inhibiting the function of miRNA. Therefore, miRNA binding site analysis of identified circRNAs is helpful for further research. The miRanda software was used to predict miRNA binding sites of the circRNAs. The most intimate top 300 pairs of combinations were screened and visualized.

### qRT-PCR to verify the expression level of target circRNA in tissues

The differentially expressed circRNA sequences were obtained by high-throughput sequencing, the screened target circRNAs were verified by real-time fluorescence quantitative PCR, and the specific primers of circRNAs were shown in Table 2, with U6 as the internal reference gene. The purity and concentration of the samples were determined by UV spectrophotometer, and if the OD260/280 values were in the range of 1.8–2.1, the RNA purity was acceptable. The cDNA was synthesized by reverse transcription using EasyScript® One-Step gDNA Removal and cDNA Synthesis SuperMix kit (TransGen Biotech, Beijing, China). cDNA was used as the template for qRT-PCR using TB Green® Fast qPCR Mix kit to detect the expression of target circRNA(Takara, Dalian, China). It was performed in a 20 μL reaction system on a real-time fluorescent PCR instrument (ABI, USA). The reaction system was 20 μL: cDNA template 5 μL, upstream and downstream primers 0.5 μL each, 2 × SYBR Green PCR Master Mix 10 μL, ddH2O 4 μL. PCR reaction conditions: 95 °C for 5 min; 95 °C for 10 s, 60 °C for 34 s, total 40 cycles. The above experiments were repeated at least three times, and statistical processing was performed using SPSS25.0 software.

**Table 2:** A qRT-PCR primer sequence information.

Gene name	Primer sequence
U6	Forward: 5′-CTCGCTTCGGCAGCACA-3′
	Reverse: 5′-AACGCTTCACGAATTTGCGT-3′
hsa_circ_0005692-	Forward: 5′-AGTGCCCAGTGCCTCGGTTC-3′
	Reverse: 5′-CGCCCGGAGCTTGGTGATAC-3′

### Statistical analyses

Statistical analyses were performed with SPSS 25.0. All the data are displayed as the mean SD for triplicate independent measurements. Student's t-test was used to assess the differences between experimental groups. Differences with P-values < 0.05 were considered statistically significant.

### Ethics approval and consent to participate

This study was approved by the Second Affiliated Hospital of Nanchang University (Nanchang, China). All procedures performed in this study using human data were in accordance with the Declaration of Helsinki (as revised in 2013). And written informed consents were obtained from each patient Data Availability Statement.

## Results

### CircRNAs identification

**Figure 1:**
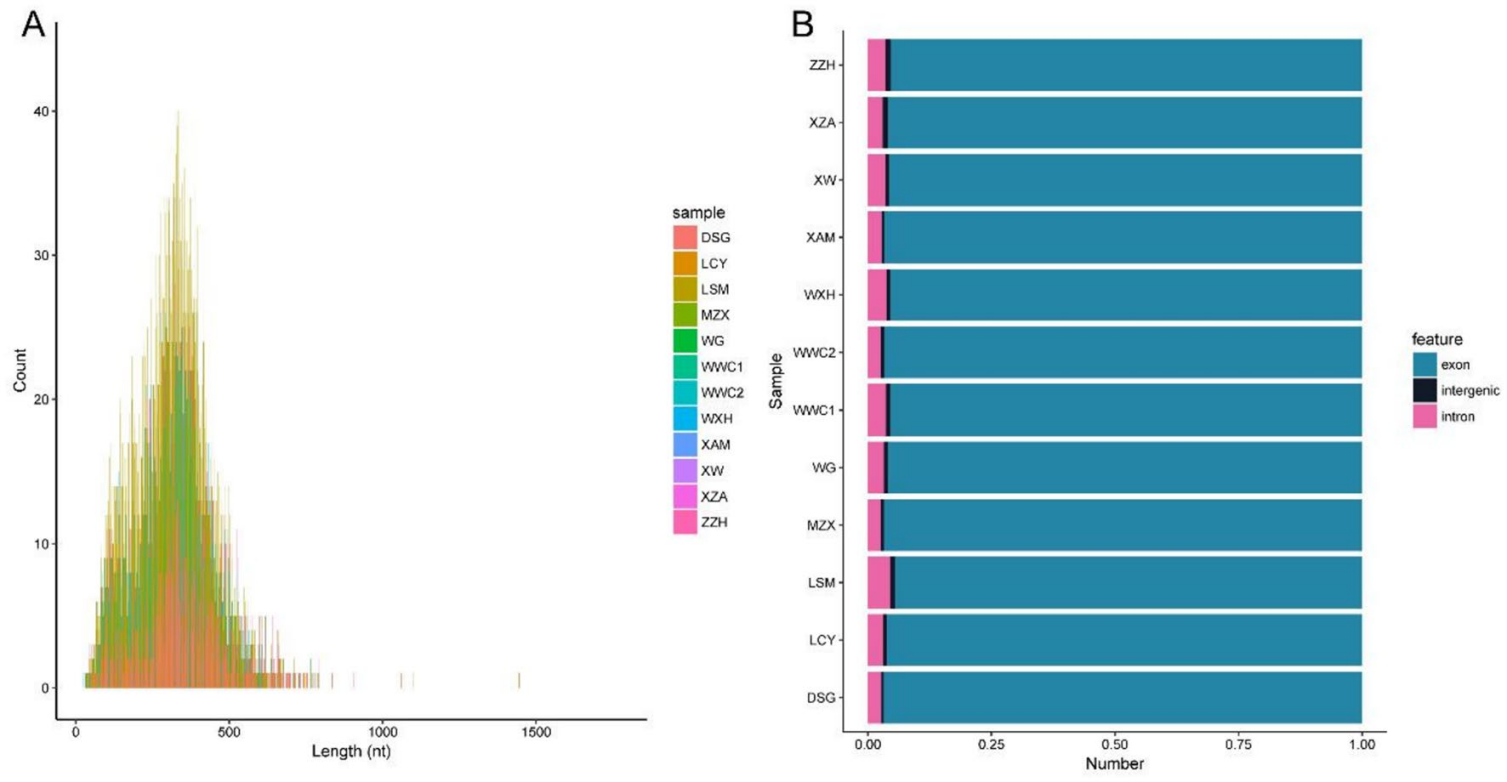
Basic information of circRNA. (**A**) CircRNA length distribution, horizontal coordinate: circRNA length; vertical coordinate: frequency of circ of that length. (**B**) CircRNA source statistics histogram, horizontal coordinate: sample name; vertical coordinate: percentage of circRNA source types.

In this study, find circ and CIRI were selected to jointly identify circRNAs. CircRNA expression profiles were screened by high-throughput sequencing technology, and a total of 13,047 circRNAs were identified through analysis of sequencing data (Additional file 1) and the lengths of the identified circRNA fragments are detailed in Fig. 1A. From Fig. 1A, it can be found that almost all the circRNAs were less than 1000 nt in length, and about 90% of them were less than 500 nt in length. In Fig. 1B, the source statistics of the circ-RNAs were obtained from sequencing, among which about 95% were derived from exons, about 3% were derived from intervenes, and roughly 1% were derived from intervenes.

### Screening of differentially expressed circRNAs

**Figure 2:**
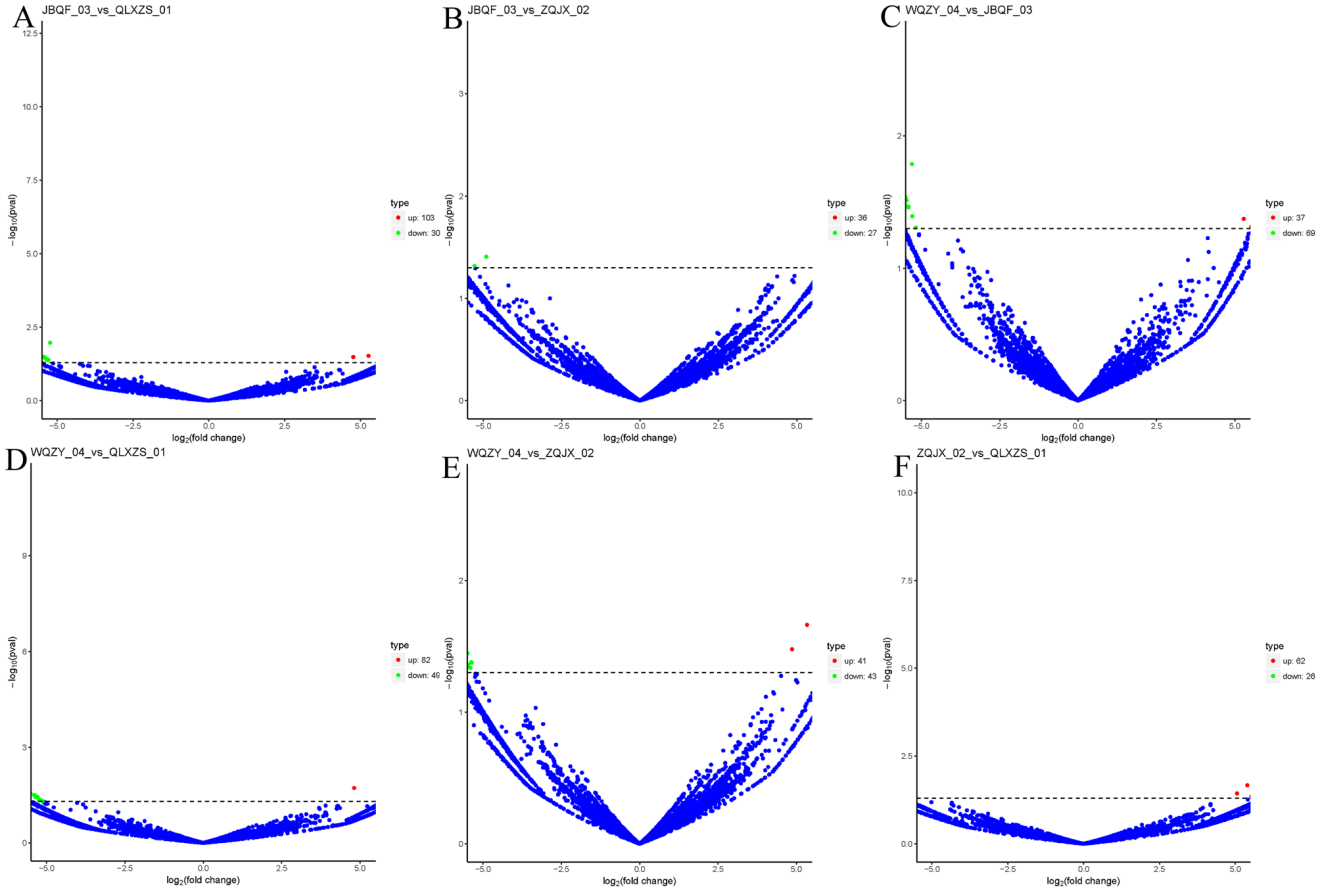
Volcano plot of differentially expressed circRNAs, horizontal coordinates represent circRNA expression fold change (log2FoldChange), vertical coordinates represent the degree of circRNA expression change, red dots indicate circRNAs whose expression levels are significantly upregulated and statistically significant, green dots indicate circRNAs whose expression levels are significantly downregulated and statistically significant. Blue dots indicate circRNAs with no statistical significance. (**A**) Local invasion vs. prostatic hyperplasia (**B**) Local invasion vs. early restriction (**C**) Late metastasis vs. local invasion (**D**) Late metastasis vs. prostatic hyperplasia (**E**) Late metastasis vs. early restriction (**F**) Early restriction vs. prostatic hyperplasia.

**Figure 3:**
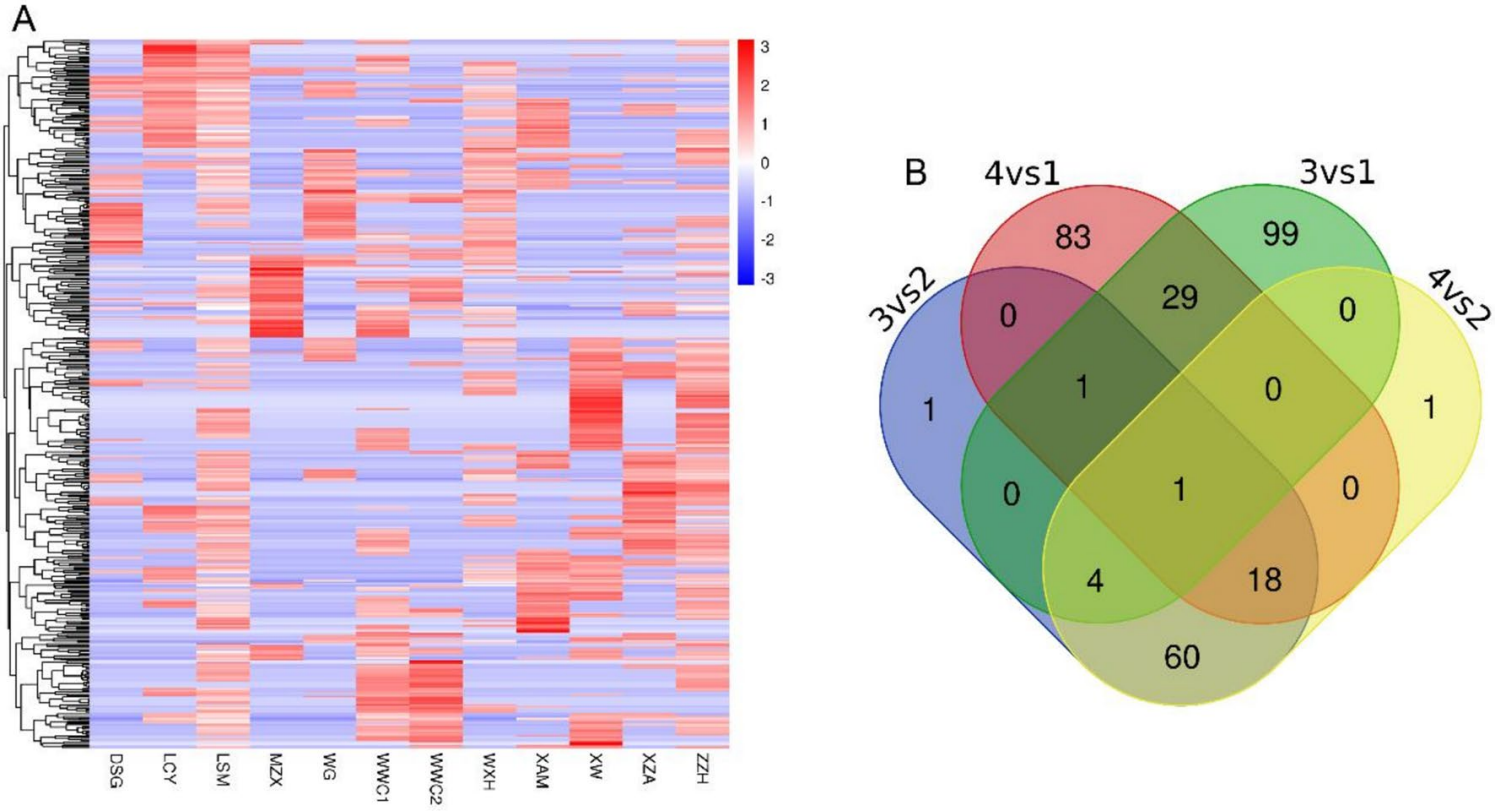
Heatmap and Venn diagrams of differentially expressed circRNAs. (**A**) Heat map of differential expressed circRNA expression. The values of circRNA expression are indicated by the color scale; from red to blue indicates the importance of the word gradually decreases, each column represents a tissue sample, and each row represents one circRNA. The results show that the data can distinguish prostate tissue from prostate cancer patients and BPH patients, and the data are logical. (**B**) Venn diagrams of differentially expressed circRNAs. 1: prostate hyperplasia group; 2: early-stage limited group; 3: local progression group; 4: advanced metastasis group.

The study used DEGseq software to analyze the differential expression of circRNAs, and the differentially expressed circRNAs were screened according to the screening conditions: fold change (FC) of gene expression values > 2 or FC < − 2, p-value < 0.05, to pick out the significantly differentially expressed circRNAs for the subsequent study. By comparing each group one by one, 605 differentially said circRNAs were screened, of which 361 circRNAs were up-regulated considerably, and 244 circRNAs were significantly down-regulated (Additional file 2 and Additional file 3). The data of the significantly differentially expressed circRNAs were made into a Volcano plot (Fig. 2A–F) and an expression heatmap (Fig. 3A). The number of differentially expressed circRNAs in each group was counted (Additional file 4), and a Wayne plot was made to visualize the circRNAs common to each group (Fig. 3B). Only one circRNA was found to be abnormally expressed in the common interval of each group. Compared with the circbase database, this circRNA was named hsa_circ_0005692 in circbase. hsa_circ_0005692 was downregulated up to about 30-fold compared with BPH tissue. hsa_circ_ 0005692 is a circRNA of antisense strand origin on chromosome 16, with a specific genomic position of chr16:4382215–4383520, containing 1305 bases. Taken into account, hsa_circ _0005692 was finally selected as our target circ RNA.

### Differential circRNA source gene GO enrichment analysis and KEGG analysis

**Figure 4:**
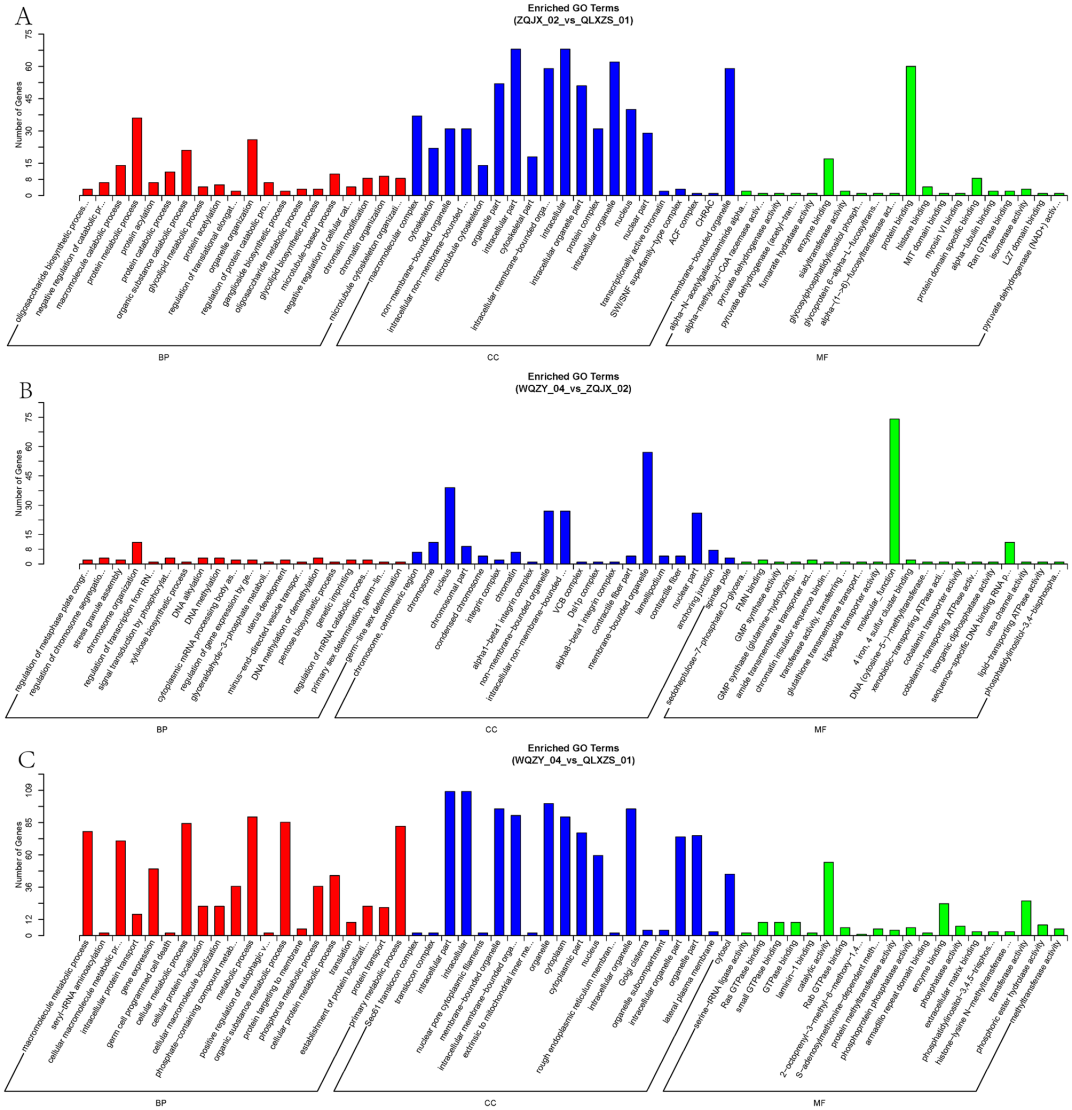
GO enrichment histograms of differential circRNA-derived genes for the first three comparison groups. (**A**) Early limited group vs. Prostate hyperplasia group. (**B**) Advanced metastasis group vs. Early limited group. (**C**) Advanced metastasis group vs. Prostate hyperplasia group. The horizontal coordinates are the GO terms at the next level of the three major GO categories, and the vertical coordinates are the number of source genes annotated to the term (including the subterms of the term) and their number in relation to the total number of annotated source genes. *GO* Gene ontology, *BP* biological process, *CC* cellular component, *MF* molecular function.

**Figure 5:**
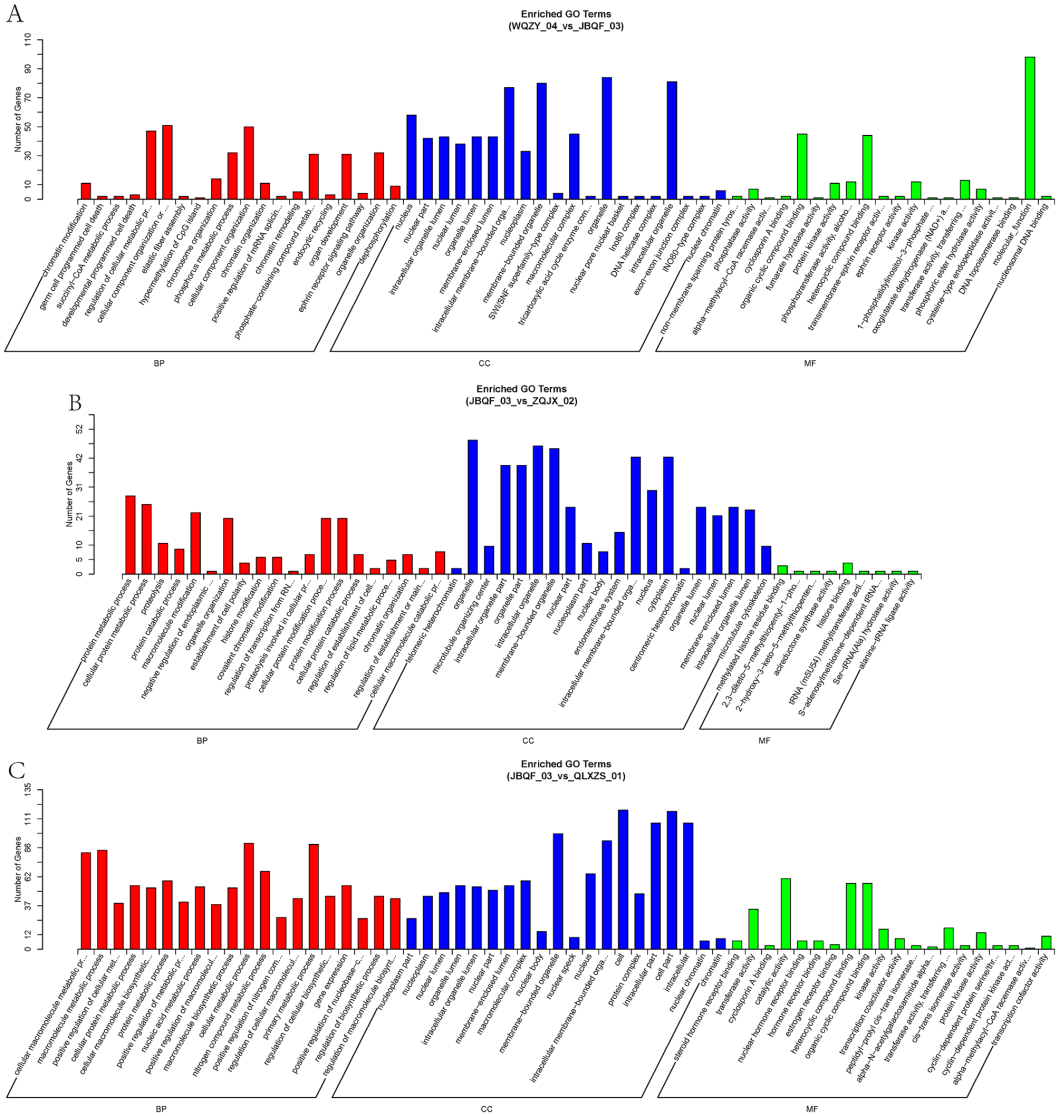
GO enrichment histograms of differential circRNA-derived genes for the latter three comparison groups. (**A**) Advanced metastasis group vs. Local progression group. (**B**) Local progression group vs. Early limited group. (**C**) Local progression group vs. Prostate hyperplasia group. The horizontal coordinates are the GO terms at the next level of the three major GO categories, and the vertical coordinates are the number of source genes annotated to the term (including the subterms of the term) and their number in relation to the total number of annotated source genes. *GO* Gene ontology, *BP* biological process, *CC* cellular component, *MF* molecular function.

**Figure 6:**
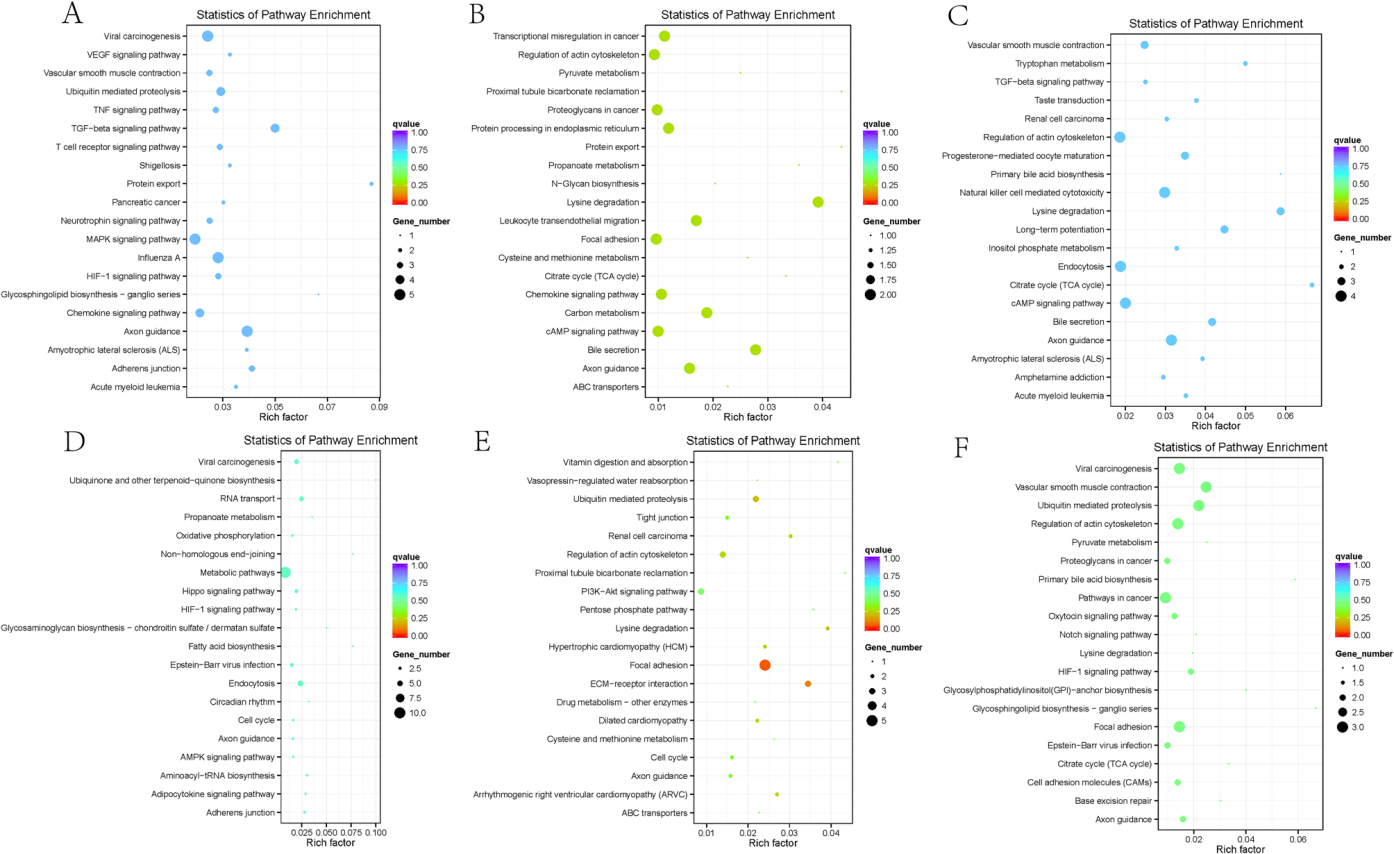
KEGG scatter plot of differentially expressed circRNAs source genes. The vertical axis indicates the pathway name, the horizontal axis indicates the Rich factor, the size of the dots indicates the number of source genes in this pathway, and the color of the beads corresponds to different padj ranges. (**A**–**F**) The KEGG scatter plot of differentially expressed circRNAs source genes for each group.

GO functional enrichment analysis and KEGG analysis were performed on the source genes of the differentially expressed circRNAs to indirectly predict the functions of circRNAs, and the 20 most significantly enriched mappings were selected. The five most significant biological functional processes analysed by GO enrichment are the regulation of cellular metabolic processes, regulation of primary metabolic processes, metabolic processes of organic substances, and regulation of metabolic processes of macromolecules (Figs. 4A–C, 5A–C). KEGG enrichment analysis revealed that the three main channels are the focal adhesion kinase FAK pathway, the extracellular matrix receptor (ECM-receptor interaction) channel, and the ubiquitin-mediated protein degradation pathway (Fig. 6A–F). These play an essential role in tumorigenesis, development, metabolism, and transcription.

### Analyse circRNA-miRNA-Netzwerken

**Figure 7:**
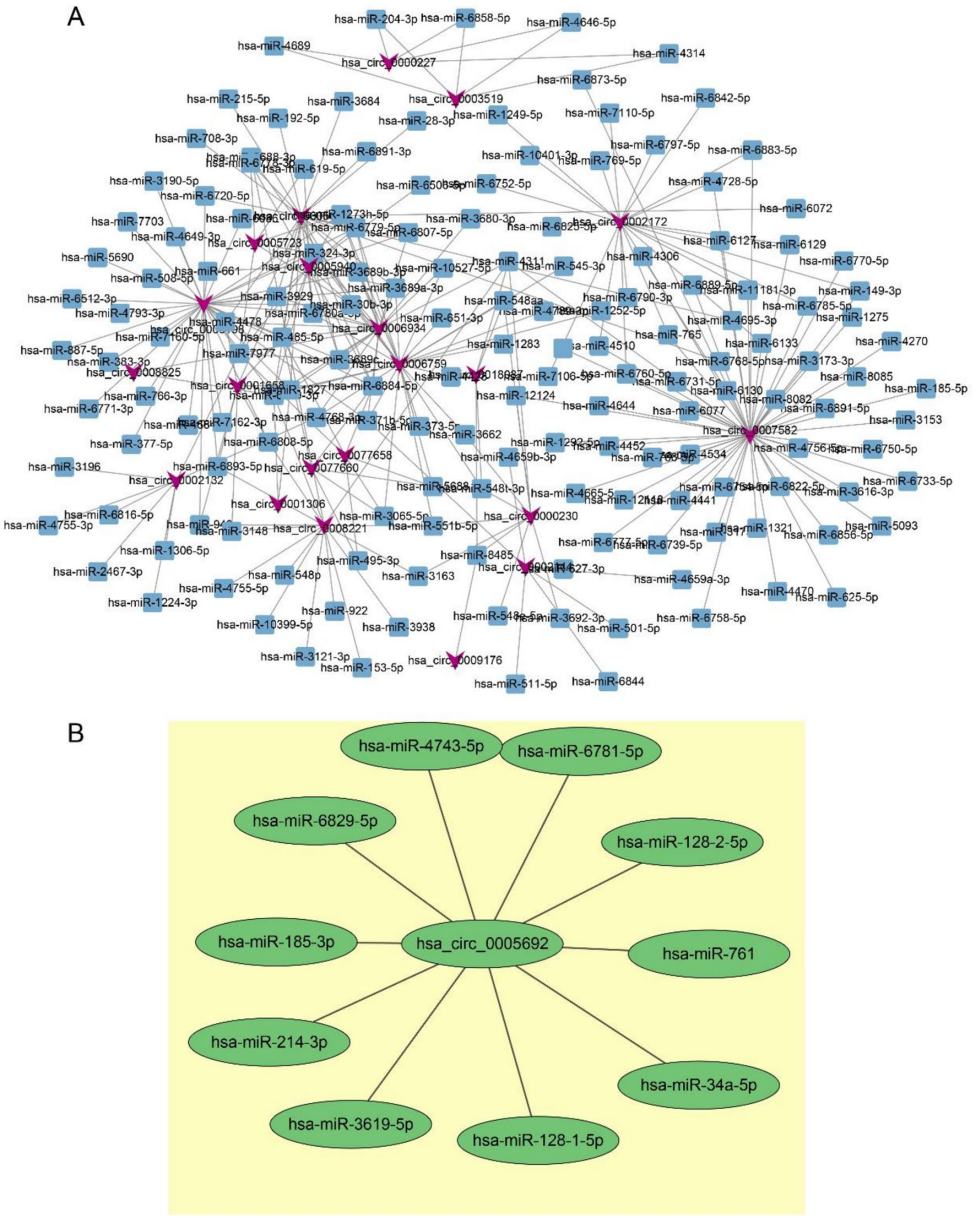
Two network regulatory maps of miRNA–circRNA. (**A**) MiRNA–circRNA regulatory network diagram. (**B**) Binding site network of miRNA of hsa_circ_0005692.

The total score of all binding sites was predicted based on the targeting relationship between circRNAs and miRNAs, and the top 300 pairs of combinations were screened and visualized with the help of tools such as Cytoscape (Fig. 7A). As shown by the circ-miRNA regulatory network diagram, the top 5 differentially expressed circRNA binding miRNAs were: hsa_circ_000 7582 (52), hsa_circ_000 6198 (37), hsa_circ_000 6759 (28), hsa_circ_000 5675 (25), hsa_circ_000 2172 (22).

### Regulatory network of hsa_circ_0005692 and miRNA

Using Miranda software to predict the binding sites of miRNAs for hsa_circ_0005692, the top 10 miRNAs in terms of binding capacity were hsa-miR-3619-5p, hsa-miR-6781-5p, hsa-miR-128-1-5p, hsa-miR-4743-5p, hsa-miR-185-3p, hsa-miR-214-3p, hsa-miR-128-2-5p, hsa-miR-761, hsa-miR-6829-5p, hsa-miR-34a-5p, and each miRNA had two or more binding sites (Fig. 7B). It is suggested that hsa_circ_0005692 may competitively bind these target miRNAs and thus further perform the corresponding functions to regulate upstream source genes or downstream target genes.

### Results of qRT-PCR

**Figure 8:**
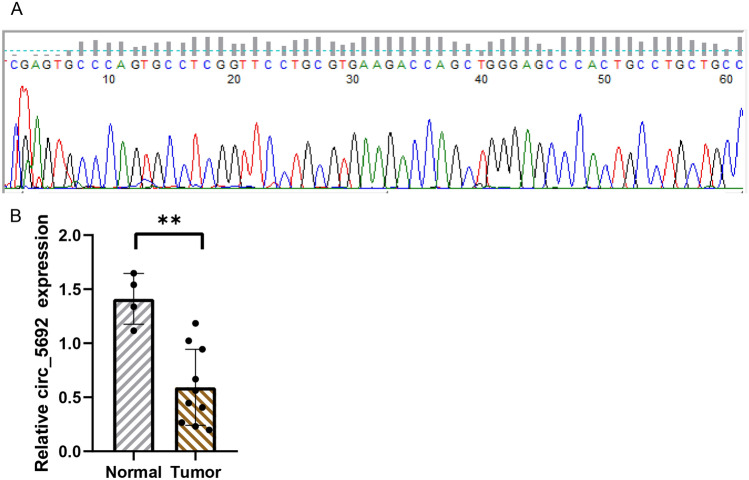
A qRT-PCR validation. (**A**) Sanger sequencing of PCR products. (**B**) Results of qRT-PCR validation of hsa_circ_0005692 expression both in normal and tumor tissues.

Sanger sequencing was performed on the PCR products, and it was seen that the results were consistent with the circbase database, indicating the specificity of the primers (Fig. 8A). qRT-PCR validation of hsa_circ_0005692 revealed a significantly lower expression level in prostate cancer compared with that in the BPH group, which was fully consistent with the results in high-throughput sequencing (Fig. 8B). It indicated that the accuracy and reliability of the results of this high-throughput sequencing were high.

## Discussion

CircRNAs are widely found in eukaryotes and are a class of abundantly expressed, highly tissue-specific, and stable non-coding RNAs that have an essential role in regulating gene expression^12^. The abnormal expression of circRNAs has been reported in esophageal squamous cell carcinoma^13^, breast carcinoma^14^, gastric cancer^15^, and hepatocellular carcinoma^16^, which are closely related to the occurrence and development of tumors. Studies on the mechanism of circRNA in prostate cancer have also emerged in recent years; Zhang et al. found that five circRNAs play an essential role in prostate cancer^17^; Hansen et al. analyzed differentially expressed circRNAs in prostate cancer and prostate cancer hyperplasia tissues and predicted the biomarker potential in prostate cancer and its close association with cancer^18^. However, few studies have been reported on the mechanisms associated with circRNAs in different stages of prostate cancer.

In this study, we constructed the expression profiles of circRNAs in prostate cancer tissues and BPH tissues by high-throughput sequencing. A total of 13,047 circRNAs were identified. 605 differentially expressed circRNAs were screened according to the screening criteria: fold change (FC) > 2 or FC < − 2, p < 0.05, of which 361 circRNAs were up-regulated and 244 circRNAs were down-regulated. Then five significant biological functional processes were analyzed by GO enrichment of the source gene collection of differential circRNAs: regulation of cellular metabolic processes, regulation of primary metabolic processes, metabolic processes of organic substances, and regulation of metabolic processes of macromolecular substances, which play an important role in the metabolic and transcriptional processes of tumors. And the main three channels that can be analyzed by KEGG enrichment are the adherent spot kinase (Focal adhesion kinase FAK) pathway, the extracellular matrix receptor (ECM-receptor interaction) channel, and the ubiquitin-mediated protein degradation pathway, and these have immediate relevance to tumor production, progression, and metastasis^19^. CircRNA can function as a sponge for miRNAs. By targeting miRNA loci analysis of circRNAs with significant differential expression, the top 5 binding miRNAs were the most abundant: hsa_circ_000 7582 (52), hsa_circ_000 6198 (37), hsa_circ_000 6759 (28), hsa_circ_000 5675 (25), hsa_circ_000 2172 (22).

Bioinformatic analysis revealed that hsa_circ_0005692 was significantly differentially expressed between groups and was a circRNA common to all groups. hsa_circ_0005692 was downregulated up to 30-fold in prostate cancer compared to BPH tissue. Comparing with the cirbase database, hsa_circ_0005692 is a circRNA from antisense strand origin on chromosome 16, specific genomic position chr16:4382215–4383520, containing 1305 bases, derived from gene GLIS family zinc finger 2. Zhang et al. found that hsa_circ_0005692 was also significantly downregulated in hepatocellular carcinoma^20^. hsa_circ_0005692 expression was also downregulated by Maass et al. when screening human circRNA profiles in human clinical precursor tissues^21^. This is consistent with our results that hsa_circ_0005692 expression is significantly downregulated in prostate cancer. However, the role of hsa_circ_0005692 in prostate cancer has not been reported. To demonstrate the confidence of the sequencing results, hsa_circ_0005692 was selected for qRT-PCR validation in prostate cancer and prostate hyperplasia tissues, and the results obtained showed that hsa_circ_0005692 was significantly down-regulated in prostate cancer tissues compared with prostate hyperplasia tissues, which was entirely consistent with the results from high-throughput sequencing.

We used Miranda software to predict the binding sites of miRNAs for hsa_circ_0005692. The top 10 miRNAs in terms of binding capacity were hsa-miR-3619-5p, hsa-miR-6781-5p, hsa-miR-128-1-5p, hsa-miR-4743-5p, hsa-miR-185-3p, hsa-miR-214-3p, hsa-miR-128-2-5p, hsa-miR-761, hsa-miR-6829-5p, hsa-miR-34a-5p, and each miRNA has 2 or more binding sites. It is suggested that hsa_circ_0005692 may competitively bind these target miRNAs to further regulate upstream source genes or downstream target genes accordingly. Taking hsa_circ_0005692/hsa-miR-214-3p as an example, Xinhua et al. revealed that miR-214-3p overexpression promotes bone metastasis and leads to poor prognosis^22^. Zhou T et al. identified RNF8 as a tumor suppressor gene, and RNF8 downregulated AR (androgen receptor) to inhibit the progression of advanced prostate cancer^23^. And one study confirmed that RNF8 is a target gene of miR-214-3p^24^. In the present study, hsa_circ_0005692 was found to have more than two binding sites to miR-214-3p, i.e., hsa_circ_0005692 could act as a "sponge" for miR-214-3p. Therefore, it can be predicted that hsa_circ_0005692/miR-214-3p/RNF8 may serve as one of the competing endogenous RNA (ceRNA) networks in prostate cancer, and it is of great significance to study the function and role of hsa_circ_0005692 in prostate cancer.

In summary, this study investigated the differences in the expression profiles of circRNAs in prostate tissues of prostate cancer patients with different clinical stages and BPH patients. And preliminary functional prediction and analysis were performed for the screened differentially significantly expressed circRNAs. Previous study has identified that hsa_circ_0005692 was downregulated in hepatocellular carcinoma (HCC), and its role in regulating HCC was rarely investigated^20^. In our study, we found that hsa_circ_0005692 was one of the markers to differentiate prostate cancer from prostate hyperplasia, it may serve as a novel diagnostic marker and target for treatment of PCa patients. In the follow-up study, we first need to expand the sample size for validation; secondly, for monitoring the development and progression of cancer including bladder cancer^25^, prostate cancer, etc.^26,27^.This study used tissue samples, and in the future, we need to find more easily available non-invasive clinical samples, such as urine, blood, etc. We aim to provide a theoretical basis for the biological function and molecular mechanism of circRNA in prostate cancer, and provide new research ideas for early diagnosis and treatment of prostate cancer.

## Conclusion

In this study, we investigated the expression profiles of circRNAs in prostate tissues of prostate cancer patients with different clinical stages and BPH patients and the preliminary functional prediction and analysis of differentially significantly expressed circRNAs, and predicted hsa_circ_0005692 as a potential new biomarker for prostate cancer. To add a new dimension to the study of the molecular mechanism of prostate cancer, and will also provide a theoretical basis for the diagnosis and treatment of prostate cancer.

### Supplementary Information

Supplementary Tables.

## Data Availability

The data generated during this study is available from the corresponding author on reasonable request. The circRNA-seq data supporting the findings of this study have been deposited in the NCBI Gene Expression Omnibus (accession GSE212215). To review GEO accession GSE212215: Go to https://www.ncbi.nlm.nih.gov/geo/query/acc.cgi?acc=GSE212215. Enter token ohwnmeycrhkjjqd into the box.

## Abbreviations

PSA: Prostate specific antigen
miRNA: MicroRNA
circRNA: Circular RNA
BPH: Benign prostate hyperplasia
GO: Gene ontology
KEGG: Kyoto encyclopedia of genes and genomes
qRT-PCR: Quantitative real-time PCR
PCa: Prostate cancer
FC: Fold change
OD: Optical density
RNA-seq: RNA sequencing
