# Supplementary material for: Identification of differentially expressed circRNAs in prostate cancer of different clinical stages by RNA sequencing
Source: Sci Rep. 2023 Dec 1;13:21175. doi: 10.1038/s41598-023-48521-7 (PMC10692156; doi:10.1038/s41598-023-48521-7)
Supplement: Supplementary file 1 — Supplementary Tables. [file 41598_2023_48521_MOESM1_ESM.pdf]

Supplementary Information

Additional file 1 CircRNA Identification Results Presentation (partial)

circRNA_ID	Chr	start	end	strand	full_length	spliced_length
hsa_circ_0005704	20	10556230	10560820	+	4590	211
hsa_circ_0002001	20	13559007	13580981	-	21974	272
hsa_circ_0004580	20	13559007	13587370	-	28363	393
hsa_circ_0059475	20	13580896	13630152	-	49256	562
hsa_circ_0006704	20	17952586	17954117	-	1531	246
hsa_circ_0009173	20	17956932	17961326	-	4393	4393

Continued Table CircRNA Identification Results Presentation

gene_id	feature	samples	Junction-read	non_junction_read	source_gene_name
**ENSG00000149346;**	**exon:10556231-10556320, exon:10557319-10557426, exon:10560700-10560820**	**LSM,MZX,WG, WWC1,WXH,XAM,XZA,ZZH**	**5,4,40,31,14,4,11,12**	**4,4,11,7,3,8,2,7**	**SLX4IP**
**ENSG00000089123;**	**exon:13559008-13559114, exon:13569507-13569586, exon:13580897-13580981**	**LSM,WWC1,WWC2**	**6,9,17**	**97,78,84**	**TASP1**
**ENSG00000089123;**	**exon:13559008-13559114, exon:13569507-13569586, exon:13580897-13580981**	**LSM,WWC1,WWC2,WXH,XAM,XW,XZA,ZZH**	**33,8,12,35,4,8,8,15**	**48,62,50,30,9,15,11,42**	**TASP1**
**ENSG00000089123;**	**exon:13580897-13580981**	**LCY**	**10**	**52**	**TASP1**
**ENSG00000089006;**	**exon:17953996-17954117**	**MZX**	**2**	**10**	**SNX5**
**ENSG00000089006;**	**exon:17961140-17961326**	**ZZH**	**2**	**2**	**SNX5**

circRNA_ID: circRNA ID (2) Chr: chromosome number (3) start: start site of circRNA full length (4) end: stop site of circRNA full length (5) strand: positive and negative strand (6) full_length: circRNA full length (7) spliced_ length (8) gene_id: circRNA source gene id (9) feature_id: circRNA splice information (for non-degenerate RNA libraries, this splice information may contain linear RNAs matching at this position) (10) samples: samples containing this circRNA (separated by commas)) (11) junction_read: number of junction reads (order corresponds to samples) (12) non_junction_read: number of non-junction reads (order corresponds to samples) (13) source_gene_name: circRNA source gene name.

Additional file 2 Statistics of the number of differentially expressed circRNAs between groups

GROUP	DIFF	UP	DOWN
Focal progression vs. BPH	133	103	30
Focal progression vs. early restriction	63	36	27
Late metastasis vs. localized progression	106	37	69
Late metastasis vs BPH	131	82	49
Late metastasis vs early limitation	84	41	43
Early restriction vs BPH	88	62	26

Additional file 3 Results of the analysis of the top 5 most significantly differentially expressed circRNAs (partial)

Groups	UP	DOWN
Locally progressive vs.BPH	**novel_circ_0009181novel_circ_0013897**	**hsa_circ_0005692 hsa_circ_0001722**
	**hsa_circ_0000377 hsa_circ_0000417**	**hsa_circ_0002275 hsa_circ_0002496**
	**hsa_circ_0000787 hsa_circ_0001673**	**hsa_circ_0003984 hsa_circ_0004872**
	**hsa_circ_0002064 hsa_circ_0002079**	**hsa_circ_0004888 hsa_circ_0005199**
	**hsa_circ_0002115 hsa_circ_0002160**	**hsa_circ_0007456 hsa_circ_0007884**
Locally progressive vs early limited	**hsa_circ_0001944 hsa_circ_0002079**	**hsa_circ_0000646 hsa_circ_0002059**
	**hsa_circ_0003247 hsa_circ_0003327**	**hsa_circ_0003027 hsa_circ_0003115**
	**hsa_circ_0003438 hsa_circ_0004535**	**hsa_circ_0003451 hsa_circ_0003842**
	**hsa_circ_0004594 hsa_circ_0006219**	**hsa_circ_0005692 hsa_circ_0005857**
	**hsa_circ_0006646 hsa_circ_0006747**	**hsa_circ_0006281 hsa_circ_0007606**
Late metastasis vs localized progression	**hsa_circ_0000302 hsa_circ_0000612**	**hsa_circ_0000371 hsa_circ_0001630**
	**hsa_circ_0001292 hsa_circ_0001730**	**hsa_circ_0002382 hsa_circ_0002762**
	**hsa_circ_0002554 hsa_circ_0002748**	**hsa_circ_0002826 hsa_circ_0003131**
	**hsa_circ_0003027 hsa_circ_0003113**	**hsa_circ_0003247 hsa_circ_0003340**
	**hsa_circ_0003962 hsa_circ_0004047**	**hsa_circ_0003915 hsa_circ_0004062**
Late metastasis vs BPH	**hsa_circ_0004961 hsa_circ_0000295**	**hsa_circ_0005692 novel_circ_0014897**
	**hsa_circ_0000343 hsa_circ_0001292**	**hsa_circ_0000495 hsa_circ_0001289**
	**hsa_circ_0001555 hsa_circ_0001726**	**hsa_circ_0002074 hsa_circ_0002275**
	**hsa_circ_0001730 hsa_circ_0002554**	**hsa_circ_0002321 hsa_circ_0002496**
	**hsa_circ_0003522 hsa_circ_0003574**	**hsa_circ_0002762 hsa_circ_0003020**
Late metastasis vs early limitation	**hsa_circ_0000234 hsa_circ_0000439**	**hsa_circ_0000073 hsa_circ_0000137**
	**hsa_circ_0001555 hsa_circ_0001726**	**hsa_circ_0000640 hsa_circ_0002844**
	**hsa_circ_0001730 hsa_circ_0002078**	**hsa_circ_0003175 hsa_circ_0003653**
	**hsa_circ_0002554 hsa_circ_0002883**	**hsa_circ_0003842 hsa_circ_0005395**
	**hsa_circ_0003307 hsa_circ_0003522**	**hsa_circ_0005692 hsa_circ_0006358**

Early restriction vs BPH	**novel_circ_0013897 hsa_circ_0000640**<br>**hsa_circ_0000958 hsa_circ_0001292**<br>**hsa_circ_0001673 hsa_circ_0002162**<br>**hsa_circ_0002607 hsa_circ_0003430**<br>**hsa_circ_0003653 hsa_circ_0003700**	**hsa_circ_0000105 hsa_circ_0000234**<br>**hsa_circ_0000495 hsa_circ_0001725**<br>**hsa_circ_0004945 hsa_circ_0007717**<br>**hsa_circ_0008775 hsa_circ_0022168**<br>**hsa_circ_0024657 hsa_circ_0035958**

Additional file 4CircRNAs common to all groups

group	quantity	circRNA id
3vs1 3vs2 4vs1 4vs2	1	hsa_circ_0005692
3vs2 4vs1 4vs2	18	hsa_circ_0002554 hsa_circ_0003522 novel_circ_0002791 hsa_circ_0001555 novel_circ_0011628 hsa_circ_0003962 novel_circ_0006521 hsa_circ_0053028 hsa_circ_0001726 hsa_circ_0003574 novel_circ_0001897 novel_circ_0006793 hsa_circ_0004027 hsa_circ_0071037 novel_circ_0012705 hsa_circ_0001730 hsa_circ_0006213 novel_circ_0012508
3vs1 3vs2 4vs2	4	novel_circ_0004659 novel_circ_0009186 hsa_circ_0072697 hsa_circ_0079136
3vs1 3vs2 4vs1	1	hsa_circ_0005692

1 indicates the prostate enlargement group

2 indicates early limited group

3 indicates localized progression group

4 indicates advanced metastasis group
